# Genomic Rearrangement and Recombination of Porcine Circovirus Type 2 and Porcine Circovirus-Like Virus P1 in China

**DOI:** 10.3389/fvets.2021.736366

**Published:** 2021-12-20

**Authors:** Libin Wen, Kongwang He

**Affiliations:** ^1^Institute of Veterinary Medicine, Jiangsu Academy of Agricultural Sciences, Nanjing, China; ^2^Key Laboratory of Animal Diseases, Diagnostics, and Immunology, Ministry of Agriculture, Nanjing, China; ^3^Jiangsu Co-innovation Center for Prevention and Control of Important Animal Infections Diseases and Zoonoses, Yangzhou, China

**Keywords:** porcine circovirus type 2, rearrangement, recombination, porcine circovirus-like virus, porcine circovirus-like mini agent

## Abstract

Porcine circovirus type 2 (PCV2) belongs to the genus *Circovirus* of the family *Circoviridae*, and it has been associated with porcine circovirus (associated) disease (PCVD or PCVAD) in pigs. PCVAD is the generic term for a series of disease syndromes that have caused economic losses to the pig industry worldwide. Since the discovery of PCV2 in the late 1990s, the virus has continued to evolve, and novel genotypes have continued to appear. Moreover, there has been recombination between different genotypes of PCV2. This review attempts to illustrate some progress concerning PCV2 in genome rearrangement and genomic recombination with non-PCV2-related nucleic acids, particularly focusing on the porcine circovirus-like virus P1 formed by the recombination of PCV2. The presence of rearranged PCV2 genomes can be demonstrated both *in vivo* and *in vitro*, and these subviral molecules ranged from 358 to 1,136 bp. Depending on whether it has the ability to encode a protein, the agents formed by PCV2 recombination can be divided into two categories: porcine circovirus-like viruses and porcine circovirus-like mini agents. We mainly discuss the porcine circovirus-like virus P1 regarding genomic characterization, etiology, epidemiology, and pathogenesis. Further research needs to be conducted on the pathogenicity of other porcine circovirus-like viruses and porcine circovirus-like mini agents and the effects of their interactions with PCV2, especially for the porcine circovirus-like mini agents that do not have protein-coding functions in the genome.

## Background

*Circoviridae* family members have short, circular, single-stranded DNA genomes 1.7–2.0 kb length. The family *Circoviridae* is divided into two genera, *Cyclovirus* and *Circovirus*, with the most studied being porcine circovirus (PCV) and beak and feather disease viruses. The genus *Circovirus* also includes members of circoviruses that infect a variety of animals, such as avian species (canary, duck, goose, pigeon, raven, gull, finch, starling, swan, and zebra finch), fishes (European catfish, barbel), mammals (pigs, bats, canines, chimpanzees, civets, humans, minks, and rodents), and arthropods (mosquitoes, ticks) (1–11).

The viruses of the *Circoviridae* family are considered to be closely related to the members of *Nanoviridae* and *Geminiviridae* that infect plants. They all share a stem-loop structure as the origin of viral replication (Ori). Gibbs and Weiller analyzed the origin and evolution of circoviruses through bioinformatic analysis based on Rep protein amino acid sequences of *Circovirus* and *Nanoviridae*. The N-terminal amino acid sequences of nanovirus and circovirus Rep were similar, while the C-terminal amino acid sequence of PCV Rep was closely related to the RNA-binding protein (protein 2C) of vertebrate calicivirus. Therefore, the authors hypothesized that PCV originated from the recombination of plant nanoviruses and vertebrate caliciviruses. Since caliciviruses have RNA genomes, retroviruses or retrotransposons may have been involved in the recombination event (12).

PCV is a non-enveloped spherical virus with a diameter of about 17 nm, and it is the smallest known animal virus at present. Four different species have been identified: PCV1, PCV2, PCV3, and PCV4. PCV1 was originally regarded as a contaminant of the continuous porcine kidney cell line PK-15 (ATCC-CCL31) in 1974, and it is generally believed that PCV1 is not pathogenic to pigs (13–15). PCV2 is recognized as an essential etiological agent of porcine circovirus (associated) disease (PCVD or PCVAD). PCVAD includes a variety of multi-symptomatic diseases such as post-weaning multisystemic wasting syndrome (PMWS), porcine dermatitis and nephropathy syndrome (PDNS), porcine respiratory disease syndrome (PRDC), and reproductive disorders, of which PMWS is the most common disease in clinical practice. PMWS was first discovered in North America in 1991 and has since been reported in pig farms all over the world. Although the clinical symptoms of PMWS are diverse, the basic characteristics are progressive weight loss, respiratory disorders, inguinal lymph node enlargement, diarrhea, pale skin, and jaundice. In addition, PCV2 can result in immunosuppression that decreases body immune functions and can occur as a concurrent or secondary infection by other pathogens; thus, PCV2 infection has resulted in huge economic losses to the pig industry (16–20). In 2016, Palinski et al. and Phan et al. detected PCV3 from pigs with PDNS and cardiac and multi-systemic inflammation, respectively (21, 22). Subsequently, Jiang et al. inoculated pigs with the rescue virus derived from PK15 cells transfected with the infectious molecular clone of PCV3 and demonstrated that it can cause PDNS-like disease (23). In 2019, PCV4 was discovered in pigs with respiratory diseases and diarrhea, and its pathogenicity is currently unclear (24).

The genome of PCVs is very small. The genomes of PCV1 and PCV2 comprise about 1,700 nt, and there are mainly two open reading frames (ORFs). ORF1 is located on the positive strand of the genome and encodes replicases related to viral replication, including rep and rep'. Rep is transcribed from the complete ORF1 and consists of 312 amino acid residues, while Rep' is a spliced transcript with 168 residues (25). ORF2 is located on the negative strand of the genome and encodes the capsid protein (CP) of the virus, consisting of 233, 234 or more residues (26, 27). The full length of the PCV3 genome is about 2,000 nt, and the PCV4 genome has 1,770 nt. The stem-loop structure formed by all these PCV nucleotide sequences contains nine conserved nucleotide motifs (TAGTATTAC), which may be related to the origin of DNA replication of PCVs via the rolling-circle replication mode. Relatively speaking, the nucleotide sequence identity of the complete genome between PCV1 and PCV2 is 68–76%. The PCV4 genome shares 66.9% sequence identity with the mink circovirus and 43.2–51.5% sequence identity with the other PCV genomes (24).

At present, research on PCV2 is more common and in-depth, and it is still the main research focus among PCVs; however, there are difficulties encountered in PCV research. One of the difficulties is that the nucleotide mutation rate of the PCV2 genome is very high. According to the whole genome analysis of 160 PCV2 strains, the mutation rate was 1.2 × 10^−3^ substitutions/site/year (28). There are also similar reports of a mutation rate of 3.1 × 10^−3^–6.6 × 10^−3^ substitutions/site/year (29). Overall, PCV2 has a high mutation rate for DNA viruses, similar to that of many RNA viruses (30).

Although the International Committee on Taxonomy of Viruses (ICTV) does not classify viruses below the species level, PCV2 can still be classified into different genotypes. Hamel et al. amplified 438 bp fragments containing each part of ORF1 and ORF2 of PCV2 by PCR, and the fragments were then digested with restriction endonucleases *Hin*fI, *Kpn*I, *Mse*I, *Rsa*I, and *Xba*I. A total of 554 PCV2 isolates in Canada from 1997 to 1999 were classified into five genotypes: CAN-2A, 2B, 2C, 2D, and 2E, accounting for 82.8, 3.0, 9.9, 1.1, and 3.2%, respectively (31). Wen et al. used similar techniques to amplify 822 (or 823) bp fragments comprising the complete ORF2 by PCR and digestion with restriction endonucleases *Sau*3AI, *Ban*II, *Nsp*I, *Xba*I, and *Cfr*I. One hundred seventy-three PCV2 strains in China from 2001 to 2003 were classified into nine genotypes of A–I, which were 0.6, 0.6, 0.6, 5.8, 8.6, 2.3, 2.3, 60.7, and 18.5%, respectively (32). In 2018, Franzo and Segalés classified PCV2 into eight genotypes (PCV2a–PCV2h) based on three criteria: maximum intra-genotype *p*-distance of 13% (calculated on the ORF2 gene), bootstrap support values higher than 70%, and at least 15 available sequences (33). The first reported genotype of PCV2 was 2a, and now the dominant genotype of PCV2 has transitioned from 2b to 2d (33, 34). There are also reports on the classification of PCV2 at the level below the genotype. For example, PCV2a is further divided into five evolutionary branches (A–E), and the average distance was 0.0158; PCV2b is divided into three evolutionary branches (A–C), and the average distance was 0.0234 (35). Moreover, the recombination of PCV2a/b and other different PCV2 genotypes has been reported in succession, making the classification of PCV2 more complicated (36–40). In this paper, the rearrangement of the PCV2 genome and its recombination with other non-PCV2-related nucleic acids rather than with different genotypes of PCV2 are reviewed.

## Genomic Rearrangement of Porcine Circovirus Type 2

In 2011, de Villiers et al. reported that the sub-genomic molecules of TT virus, formerly belonging to the family *Circoviridae* and now belonging to the family *Anelloviridae*, appeared in cell culture and passage *in vitro*, suggesting that they may play a role in the pathogenesis of TT virus (41). Similarly, Tischer and Buhk detected the sub-genomic molecules of PCV1 in PCV1 cell culture and believed that they were defective-interfering viruses (42). Since 2012, Wen et al. have obtained 23 strains of PCV2 rearrangement molecules *in vivo* and *in vitro*. Among these, 13 strains of PCV2 rearrangement molecules such as ZJQDH1, ZJQDH2, BIV, JSTZ, JSHM, and CHIVV6-CHIVV13 were derived from pigs; ten strains such as CH-IVT1, BIV, and CH-IVT3-CHIVT11 were derived from PCV2 cell cultures, and BIV was detected from both pigs and PCV2 cell culture. The results showed that their genomes were also circular, similar to PCV2, and the nucleotide sequences were completely derived from the parent strains and did not contain exogenous nucleotide fragments, but the splicing junction sites were different. Most rearranged molecules' (18/23) splicing donor sites are characterized by two identical nucleotides such as AA, TT, GG, or CC (43–46). In addition, two PCV2 rearrangement molecules HN2-3 and HN3-1 were detected from pigs, with genome sizes of 922 and 617 nt, respectively (47). Wen has also reported that the PCV2 rearrangement molecule CH-IVT12 from PCV2 cell culture has a genome size of 1,136 nt, of which 1,104–1,136 nt were highly homologous to the reverse complement sequence of nt 1,729–1,761 of the PCV2 BF strain (48). Although the genome sizes of PCV2 rearrangement molecules discovered so far are different, ranging from 358 to 1,125 nt, they can be classified according to the presence or absence of a specific stem-loop structure and ORF1 and ORF2 and by the number and composition of amino acids encoded by ORF1 and ORF2 (Table 1).

**Table 1:** Genome characteristics of the rearranged molecules from serum samples and PK15 cells infected with full-length PCV2 genomes.

**The rearranged molecules**	**Genome lengths (nt)**	**Nucleotide positions of the splice sites**	**ORF1 (aa)**	**ORF2 (aa)**	**The conserved stem–loop structure**	**GenBank accession no**.
ZJQDH1	483	139...1425	31 (28)	113 (102)	+	JQ690759
ZJQDH2	574	99...1294	–	179 (147)	+	JQ690760
JSTZ	578	159...1350	39 (35)	192 (128)	+	JQ690758
JSHM	772	28...1025	–	232 (232)	+	JQ690761
BIV	896	174...1047	134 (40)	231 (231)	+	JX524206
CH-IVV6	475	145...1439	35 (29)	146 (100)	+	JX984577
CH-IVV7	525	91...1335	–	149 (128)	+	JX984578
CH-IVV8	572	82...1279	26 (10)	190 (151)	+	JX984579
CH-IVV9	501	131...1399	27 (26)	119 (111)	+	JX984580
CH-IVV10	631	323...1461	146 (90)	91 (91)	+	JX984581
CH-IVV11	745	396...1420	124 (114)	107 (107)	+	JX984582
CH-IVV12	458	151...1462	125 (33)	152 (90)	+	JX984583
CH-IVV13	436	91...1424	–	133 (103)	+	JX984584
CH-IVT1	605	213...1377	174 (53)	201 (118)	+	JX094503
CH-IVT3	358	.962–1319.	–	–	–	JX984585
CH-IVT4	748	.1018–1765.	–	232 (232)	–	JX984586
CH-IVT5	757	.939–1695.	–	162 (162)	–	JX984587
CH-IVT6	1,073	329...1025	142 (92)	232 (232)	+	JX984588
CH-IVT7	1,125	379...1023	115 (109)	232 (232)	+	JX984589
CH-IVT8	880	140...1029	31 (29)	232 (232)	+	JX984590
CH-IVT9	883	66...952	–	232 (232)	+	JX984591
CH-IVT10	977	238...1030	110 (62)	232 (232)	+	JX984592
CH-IVT11	1,024	191...936	65 (65)	232 (232)	+	JX984593
HN2-3	922	693...1540	225 (214)	74 (65)	+	KC415247
HN3-1	617	..5–621..	226 (190)	–	incomplete	KC415248
CH-IVT12	1,136	..3–1105..	314 (314)	–	incomplete	KT868943

*Genome lengths of the rearranged molecules and the sizes of the predicted proteins corresponding to ORF1 or ORF2 are indicated. Dots in the third column represent deleted regions, and dashes indicate the unspliced nucleotides. Numbers in parentheses indicate the numbers of amino acids showing high identity with reference PCV2 BF isolate corresponding ORFs (AF381175)*.

Regarding form, these rearrangement molecules should belong to the PCV2 defective-interfering (DI) viruses, a type of sub-genome or deletion mutants produced by complete virus replication, and most are replication-deficient. In the traditional sense, the genome of a defective virus is smaller than that of a complete virus; therefore, it can replicate more rapidly when it is co-infected with the complete virus, thereby interfering with the replication of the complete virus. There are DI viruses that can aggravate diseases caused by certain viruses, for example, members of the *Geminiviridae* (49).

## Genomic Recombination of Porcine Circovirus Type 2

**Figure 1:**
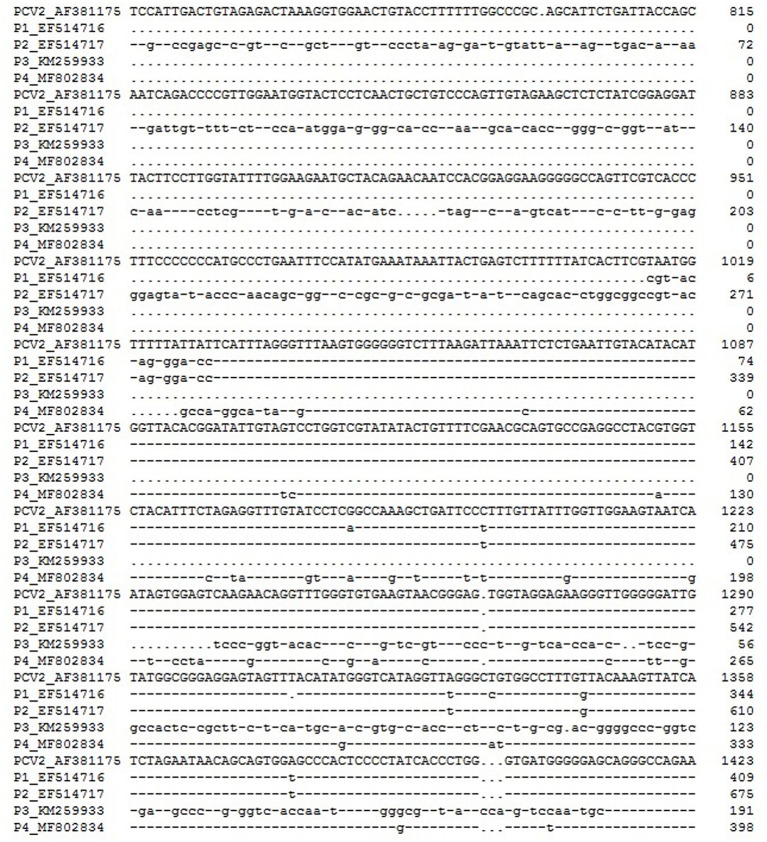
Multiple alignment of nucleotide sequences of the PCV-like viruses and PCV2. Numbers at the 3'-terminal ends indicate the length of the nucleotide sequences in each species. The full-length genome sequences of PCV2 and PCV-like viruses are not shown. The omitted 3'-terminal nucleotide sequences are highly homologous nucleotide sequences. Deletions (. dots in the sequence), identical nucleotide sequence (– dashes in the sequence).

**Figure 2:**
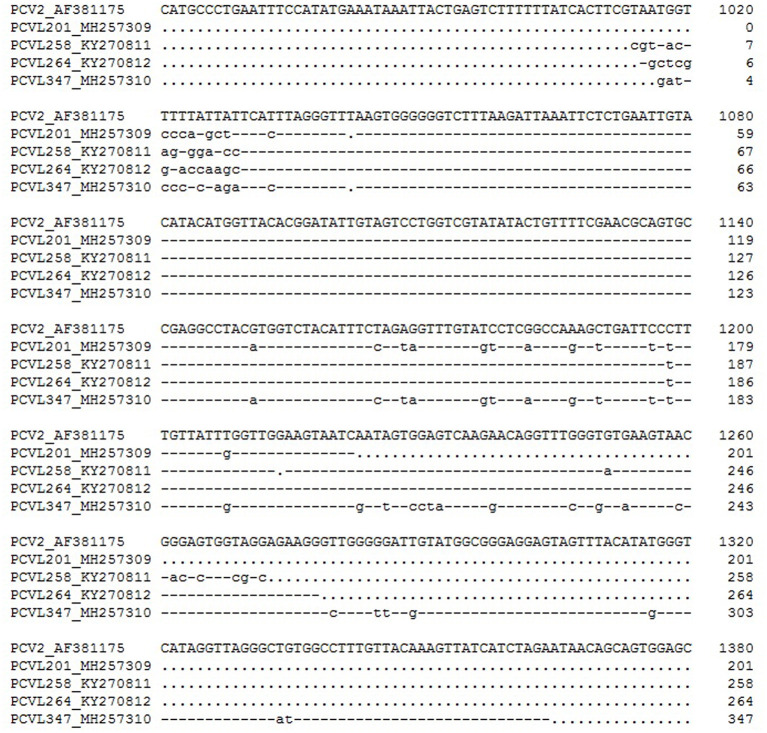
Alignment of nucleotide sequences from the PCV-like mini agents and PCV2. The full-length genome sequence of PCV2 is not shown. A dot represents a deletion, and a dash indicates an identical nucleotide in comparison with the top sequence.

The agents formed by the recombination of PCV2 can be divided into two categories: PCV-like viruses and PCV-like mini agents. The genome of the former has the ability to encode proteins, while the latter does not. Like PCV2, they also have a circular DNA genome. In addition to the high homology of most of the nucleotide sequences to PCV2, their genomes also contain small exogenous nucleotide sequences. Wen et al. have found four PCV-like viruses in pigs, tentatively named P1, P2, ZJ-R (P3), and P4. The full lengths of the viral genomes were 648, 993, 694, and 710 nucleotides, respectively. The lengths of the exogenous nucleotide fragments in the P1, P2, P3, and P4 viral genomes are 16, 281, 180, and 9 nt, respectively (Figure 1). It is speculated that P1, P2, and P4 originated from the recombination of PCV2 with other virus genomes (retrovirus, adenovirus), while P3 originated from the recombination of PCV2 with host (pig) genomes (50–53). Similar to P3, a recombinant strain HN6-1 of PCV2 with a genome size of 839 nucleotides containing a 217-nt exogenous fragment was reported; this originated from the recombination of PCV2 with pig genomes (47). Later, Wen et al. discovered four kinds of PCV-like mini agents, temporarily named PCVL258, PCVL264, PCVL201, and PCVL347. PCVL258 and PCVL264 were from pigs and were later found in dogs and cats (54, 55). Currently, PCVL201 and PCVL347 are found only in cattle. The full lengths of their circular DNA genomes are 258, 264, 201, and 347 nucleotides, containing 16-, 12-, 9-, and 11-nt exotic nucleotide sequence fragments, respectively (Figure 2). This is the first report of the agents that do not have the ability to encode proteins in infected animals (55). Interestingly, the genomes of P1, P2, and PCVL258 all contain 16 foreign nucleotide fragments (CGTTACTAGTGGATCC).

**Figure 3:**
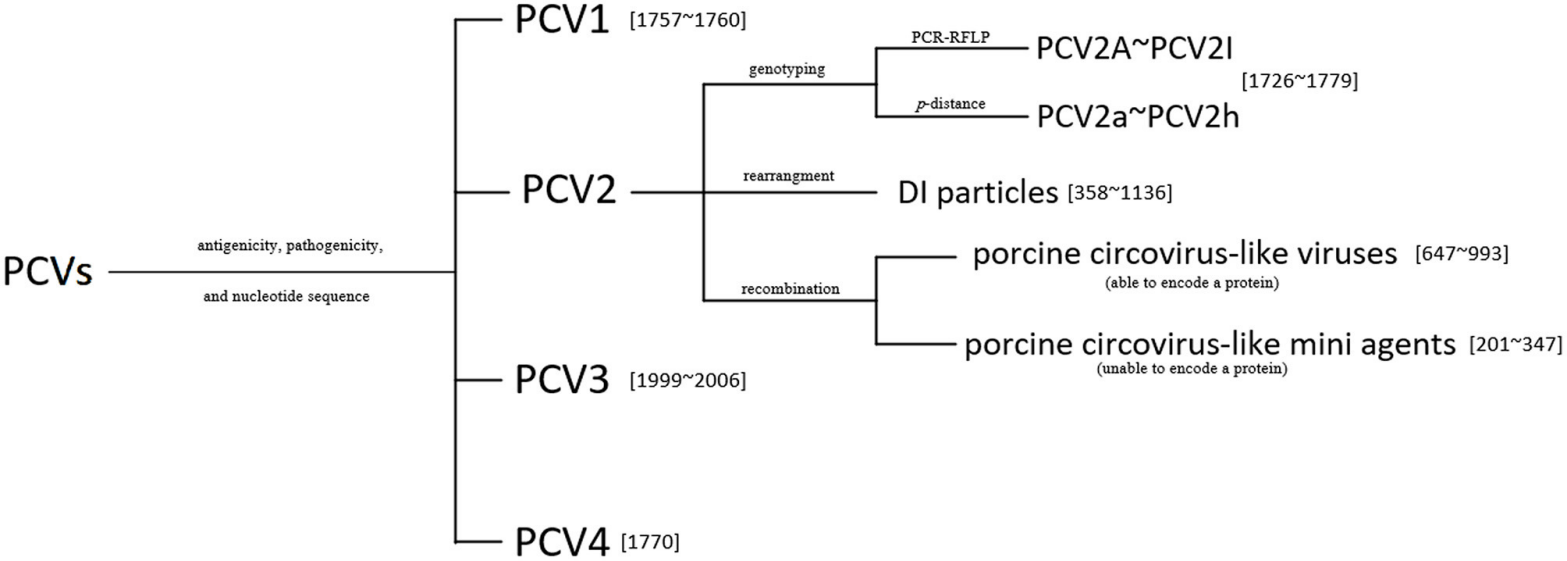
The classification of known porcine circoviruses (PCVs). The genotyping of PCV2 (A–I) using the PCR-RFLP proposed in Wen et al. (32), and the *p-*distance method for the PCV2 subtypes (a–h) proposed in Franzo and Segalés (33). Numbers in square brackets indicate the genome lengths of the viruses or the mini agents.

The current classification of PCVs can be summarized in Figure 3.

## Porcine Circovirus-Like Virus P1 in China

Since P1 is the first known PCV2 recombinant virus, and the clinical P1 detection rate was comparably higher than that of other PCV-like viruses, we carried out a more systematic study of the P1 virus.

### Genome, Gene, and Function of Porcine Circovirus-Like Virus P1

The P1 genome is a single-stranded circular DNA and is the animal virus with the smallest currently known genome. Except for 16 nucleotides that are presumed to be derived from porcine retroviruses, the remaining 632 nucleotides are highly homologous with PCV2 ORF2.

Although its genome is relatively small, eight ORFs have been verified by PCR, RACE, and Northern blotting. ORF1, ORF2, ORF4, and ORF5 are located on the negative strand of the P1 virus genome, and the positions are 151–493, 492–579, 279–327, and 1–52, respectively. ORF3, ORF6, ORF7, and ORF8 are located on the positive strand of the genome, and the positions are 494–599, 263–293, 285–336, and 374–419, respectively (56).

**Figure 4:**
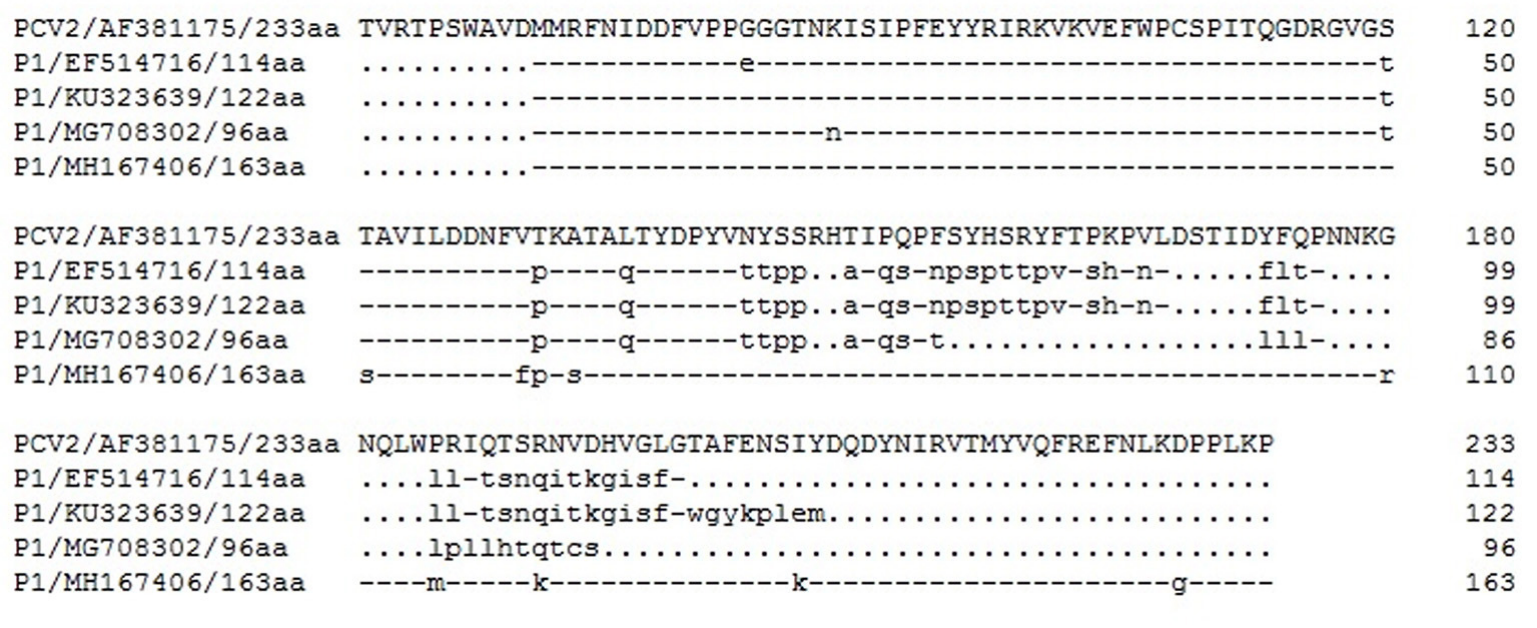
Sequence alignment of the deduced amino acid sequences of the capsid proteins of representative porcine circovirus type 2 (PCV2) and porcine circovirus-like virus P1. All strains are marked by accession no. and by amino acid length of the protein. The diagram was generated using DNAman 7.0 software. Dashes represent amino acids that are identical to those of the PCV2. Dots stand for the deleted amino acids compared with PCV2.

P1 ORF1 is the largest ORF, encoding a capsid protein of 114 amino acids with a protein size of ~12.5 kDa. Its N-terminal amino acid sequence is highly homologous to the PCV2 capsid protein sequence, while the C-terminal amino acid sequence has no homology with PCV2 capsid protein due to the deletion of one nucleotide corresponding to PCV2. The ORF1 of the original P1 strain encodes 114 amino acids, and a mutant strain appeared in 2017 with ORF1 encoding 122 amino acids (57). In 2018, a P1 strain was reported with a total length of 647 nucleotides and a G deletion at position 183 in the genome, reducing the number of amino acids encoded by ORF1 to 96 (58). Despite these mutations, phylogenetic analysis showed that these P1 strains were dispersed in a large branch until the emergence of a cat-origin strain, and the nucleotide sequence of its genome has characteristic variation that makes it another evolutionary branch (54). In 2020, three P1 strains were detected, each with a total length of 649 nucleotides. The nucleotide sequences with high homology with PCV2 had no nucleotide deletions, and ORF1 encoded 163 amino acids. Phylogenetic analysis showed that they were closely related to other P1 strains at the nucleotide level and to PCV2 at the capsid protein amino acid level, suggesting that they were an indispensable component in the evolution of PCV2 and P1 (59) (Figure 4).

Using the constructed double-copy tandem molecular cloning of whole P1 genome and DNA site-directed mutagenesis technology, the P1 molecular clones with each ORF deletion were transfected into PK15 cells, and then the total RNA was extracted 12 h later for gene chip analysis. The results showed that P1 ORF1 was involved in the biological processes of defense response to virus, signal transduction, regulation of Rab GTPase activity, and lipid metabolic process; ORF1 was involved in diverse molecular functions of protein phosphatase inhibitor activity, phosphatidylinositol phospholipase C activity, phosphoric diester hydrolase activity, and Rab GTPase activator activity, and in pathways of digestive gland secretion (gastric acid, salivary, pancreatic) and neural development (60, 61).

P1 ORF2 was involved in the biological processes of positive regulation of cell growth, proliferation and migration, positive regulation of leukocyte chemotaxis, and defense response to virus; in molecular functions, ORF2 was involved in insulin-like growth factor binding and chemokine activity, and ORF2 was involved in pathways of the cytosolic DNA-sensing pathway, RIG-I-like receptor signaling pathway, toll-like receptor signal pathway, chemokine signal pathway, and cytokine and cytokine-cytokine receptor interactions (61).

The biological processes, molecular functions, and related pathways involved in P1, ORF3, and ORF5 are basically similar to those of ORF2 (61).

P1 ORF8 is mainly involved in biological processes such as purine nucleotide biosynthetic process, amino acid transport, amino acid transmembrane transport, and defense response to virus and in molecular functions such as AMP-lyase activity and amino acid transmembrane transporter activity (61).

### Porcine Circovirus-Like Virus P1 Infectivity and P1-Associated Diseases

Both rearrangement molecules and recombinant viruses of PCV2 have been found in pigs. Except for the two PCV-like mini agents (PCVL201 and PCVL347) from the sera of clinically healthy cattle (55), all PCV2 rearrangement molecules formed *in vivo*, PCV-like viruses, and other PCV-like mini agents can be detected in the sera of PMWS cases. In addition, PCV-like mini agents (PCVL258 and PCVL264) can also be detected in the sera of dogs and cats with respiratory diseases or enteric diseases (54). However, further research is needed to offer conclusive evidence of a link between these agents and those diseases.

The reports of P1 virus infection are currently only from China. P1 infection was first detected by PCR in 248 pigs from six pig farms in Jiangsu Province, China. The results showed that three farms were P1-positive, with 19% infection rate. About 30% of the P1 antibody positives in pigs in Jiangsu, Zhejiang, Shandong, and Shanghai were detected by the indirect ELISA established by the prokaryotic expression of part of the P1 capsid protein, and the P1 infection rate of pigs with PMWS was significantly higher than that of pigs without PMWS (62, 63). P1 virus can occur alone or in coinfection with PCV2. In addition to pigs, it can also naturally infect cattle, rabbits, goats, dogs, and cats (54, 64).

PK15 cells were transfected with the double-copy tandem molecular clone of the P1 virus genome, and the inclusion bodies were found in the cytoplasm and nucleus of cells by electron microscopy. In comparisons, there was a large number of cytoplasmic inclusion bodies that were basically round, with a diameter of about 0.1–0.3 μm. There were two types of nuclear inclusion bodies: one was small and round with a diameter of about 0.1 μm and high electron density; the other was slightly hexagonal with a size of about 0.4–0.8 μm (65).

The pigs inoculated with the P1 infectious clone through inguinal lymph nodes began to develop viremia in varying degrees at day 14. Some pigs showed similar clinical symptoms as PMWS characterized by weight loss, pale skin, and diarrhea during the experiment (66). After 35 days of inoculation, there were gross lesions of encephalemia, hemorrhages of the bladder mucosa, hemorrhages of inguinal lymph nodes, and lung atrophy and lung hemorrhages in the pigs. Histological changes showed arteriectasis and telangiectasia of the cavitas subarachnoidealis, interstitial pneumonia, myocardial atrophy, histiocytic hyperplasia of the follicles in the tonsils, and necrosis of pancreatic cells. The nucleic acids and antigens of P1 were detected from the lung, brain, heart, liver, bladder, pancreas, and gonad by PCR and immunohistochemistry. The P1 rescue virus from an infectious DNA clone is a spherical, non-enveloped virus with a diameter of about 25 nm (66).

BALB/c mice were artificially infected with P1 rescue virus and divided into a single-challenge group and a three-challenge group (7-day intervals for each time). The results showed that on the 14th and 21st days after challenge, the weight gain rate of the mice in the three-challenge group was significantly lower than that in the control group. ELISA antibodies were detected throughout the 35- day experimental period. Viral nucleic acids were detected by PCR in the heart, liver, spleen, lung, bladder, testis, brain, thymus, and pancreas. Viral antigens were detected in the heart, liver, spleen, lung, testis, and thymus using immunohistochemistry. Histopathological changes of mice infected with P1 virus showed interstitial pneumonia, including perivascular connective tissue edema, mild inflammatory cell infiltration, alveolar wall thickening, myocardial necrosis, and Purkinje nucleolysis (67).

Congenital tremor (CT) in piglets is a sporadic disease characterized by paroxysmal muscle spasms. It mostly occurs in newborn piglets without seasonality. The disease was first reported in western Canada in 1992, and has since occurred in many countries. The diseased piglets are born characterized by systemic tremors. Most suffer from severe malnutrition or starvation due to lack of colostrum or colostrum deficiency. In severe cases, the mortality is as high as 100%. In general, there are no obvious changes observed in the central nervous system, peripheral nervous system, or skeletal muscle of the affected piglets through gross anatomical examination. However, different degrees of hypomyelination and mild vacuolization of the central nervous system are typical histological features (68, 69).

Previous studies have shown that CT may be caused by a classical swine fever virus named “Linda virus,” an atypical porcine pestivirus (APPV), an astrovirus, or a porcine teschovirus (70–74). Wen et al. detected 100% of P1 nucleic acids in the serum, liver, lung, brain, and inguinal lymph nodes of four pigs with CT from a pig farm in the Jiangsu province of China, and P1 nucleic acids were also detected in the heart, kidney, and pancreas of some pigs. Histopathological changes showed that various organs experienced different degrees of tissue damage and inflammatory cell infiltration. The damage to lymph node tissues was more serious; some lymphocytes in lymph follicles dissolved and disappeared. Interstitial pneumonia, pancreas with vacuoles, partial Purkinje cell loss, and nucleolysis were also observed (75).

Besides PCV-like virus P4, PCV-like virus P1 was also detected in the fetal lung tissues of unexplained abortions in pig farms in Jiangsu and Anhui provinces in 2017; these tested negative for classical swine fever virus (CSFV), porcine parvovirus (PPV), porcine reproductive and respiratory syndrome virus (PRRSV), Japanese encephalitis virus (JEV), pseudorabies virus (PRV), and PCV2 by PCR or RT-PCR (53).

Syndromes and diseases associated with P1 virus are summarized in Table 2.

**Table 2:** Clinical syndromes and diseases associated with P1 virus based on natural or artificial infection or both.

	**PMWS**	**CT**	**Respiratory**	**Enteric**	**Reproductive**
Natural infection	√	√	√	√	√
Artificial infection	PMWS-like	–	–	–	–

*(√) association; (–) no experiment yet*.

### Pathogenic Mechanism of Porcine Circovirus-Like Virus P1

Compared with the control group, there were five indicators that displayed no differences in the peripheral blood erythrocyte parameters of pigs infected with PCV-like virus P1: red blood cells (RBC), hemoglobin (HGB), hematocrit (HCT), mean corpusular volume (MCV), and mean corpuscular hemoglobin concentration (MCHC). While the mean corpuscular hemoglobin (MCH) decreased significantly at 17 days after inoculation, the red blood cell volume distribution width (RDW) increased significantly at 31 days. There was no significant difference in the mean platelet volume (MPV), but the platelet count (PLT), and plateletocrit (PCT) decreased significantly at 8 days after inoculation. The platelet volume distribution width (PDW) decreased significantly at 17 days, suggesting that P1 can lead to small-cell hypochromic anemia characterized by an abnormally low MCH in the red blood cells. Significantly more apoptosis-positive cells at days 21 and 35 after inoculation were detected in the immune organs and tissues (spleen, lymph node, and tonsil) by terminal-deoxynucleotidyl transferase mediated nick end labeling (TUNEL) assays (76–78).

In the first week after challenge with P1 virus, the MPV of Balb/c mice significantly increased compared with that of the control group. Similarly, in the fifth week after challenge, the MCH and MCHC were significantly lower than those in the control group (67).

The expression of TLRs mRNA was down-regulated at 3 dpi, except for TLR 2 and TLR 10. Since then, TLRs mRNA expression levels of different phases of infection groups were basically in recovery trends. The expression of TLR2 mRNA was significantly increased on the 24th and 40th days after infection, and the expression of TLR9 mRNA on the 31st and 40th days after infection also showed an up-regulated trend. The results suggested that the inflammatory response mediated by TLR2 and TLR9 may play an important role in P1 recognition and its pathogenesis (79).

The mRNA expression levels of cytokines IL-2, IL-4, IL-6, IL-8, IL-10, IL-12p40, and IFN-γwere relatively different after P1 infection. The mRNA of IL-4 on the 24th day after infection, IL-8 on the 24th and 40th days, and IL-12p40 on the 40th day were significantly up-regulated; IL-6 mRNA was up-regulated during the test period, while IL-10 mRNA was significantly down-regulated on the 3rd and 31st days after infection. The changes in these cytokines can preliminarily clarify that P1 infection may be characterized by excessive inflammatory response, leading to anemia (80).

On the third day after P1 infection, the expression of CD80 mRNA in porcine alveolar macrophages showed a down-regulated trend, and the expression levels of CD40, CD74, and SLA-DR were significantly down-regulated. The expression levels of SLA-DR and SLA-DM on the 8th day and SLA-DM on the 17th day showed a down-regulated trend. The expression of CD86 mRNA showed an up-regulated trend on the 24th day after infection. The expression of CD74 mRNA was significantly down-regulated at 40 dpi. The results revealed that PCV-like virus P1 infection had a significant inhibitory impact on MHC class II processing and presentation of antigens of porcine alveolar macrophages, affecting the immune response (81).

**Figure 5:**
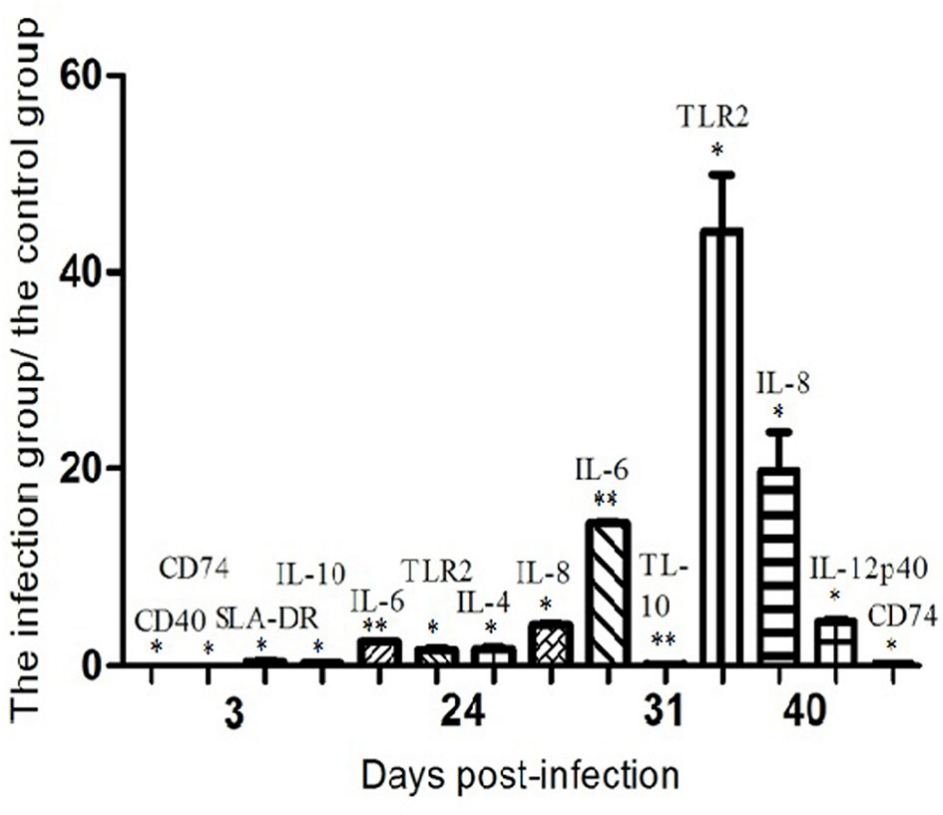
The mRNA expression profiles of functional molecules in peripheral blood mononuclear cells or alveolar macrophages from P1-infected and healthy piglets as determined by semiquantitative RT-PCR. For each molecule, the amounts of RT-PCR products were normalized to the values obtained with β-actin, used as an internal standard for each sample. The relative levels of the molecules mRNA expression are expressed as the mean fold increase of the virus-infected groups vs. control animals, error bars represent standard deviations. Statistical analysis was performed by means of the two-tailed unpaired Student's *t*-test. *P < 0.05, **P < 0.01.

The above-mentioned protein molecules with significant changes at different times after P1 virus infection are summarized in Figure 5.

The Wnt pathway is a signal pathway that exists in multicellular organisms and that has a high degree of evolutionary conservation. It is essential for the growth and development of the organism, and it can regulate the polarity and differentiation of cells. The results showed that the mRNA levels of MMP-2 and c-mys in tissues, cyclin D1 in liver, MMP-7 and MMP-9 in kidney, and DKKK-1 in lymph nodes and kidney were significantly decreased when pigs were infected with P1 virus for 5 weeks. After P1 virus infected ST cells for 24, 48, and 72 h *in vitro*, the level of MMP-2 mRNA decreased significantly; the level of c-mys mRNA decreased significantly at 24 h. The level of β-catenin protein was down-regulated; it was prevented from entering the nucleus, and TCF/LEF promoter activity was eliminated. The results showed that P1 virus infection could inhibit the Wnt pathway activity both *in vivo* and *in vitro* (82).

## Conclusions and Perspectives

PCV2 is the primary causative agent for PCVAD, it may require other factors or agents to cause PCVAD. P1 virus infection alone can lead to symptoms similar to PMWS in pigs, and it has the nucleotide sequence highly similar to PCV2, these characteristics will make P1 virus a breakthrough in understanding the relationship between PCV2 and PMWS, although P1 virus has been reported by one research group only and has not attracted much attention.

It has been 30 years since PCV2 was first reported, and recent evidence shows that PCV2 has circulated in pig herds for a long time. In the evolution of PCV2, the emergence of new genotypes coupled with the rearrangement and recombination resulting in the formation of dozens of pathogenic molecules or agents makes the diagnosis of PCV and the prevention and control of PCVD difficult. At present, some of the PCV2 rearrangement molecules have the ability to encode relatively complete ORF1 and ORF2 at the same time, and some completely lack ORF1 or ORF2. Some ORF1 and ORF2 express proteins with different C-terminal amino acid sequences from PCV2 ORF1 and ORF2 due to splicing rearrangement. Therefore, whether they can replicate and express proteins by themselves, and how they affect the replication and pathogenicity of PCV2 remain to be studied in depth. The genomes of the PCV-like viruses formed by PCV2 recombinants are mostly <1,000 nt in length; these are the smallest currently known viral genomes. Compared with PCV2, there is no ORF encoding a putative replicase protein in the genomes of these PCV-like viruses, and how they replicate needs to be clarified. Besides P1 virus, other PCV-like viruses, especially P3 formed by the recombination of PCV2 and host genomes, need to be further studied whether they are pathogenic to pigs. In particular, these two characteristics of the small circular genomes and the lack of the ability to encode proteins make the PCV-like mini agents similar to the viroid genome, which is only a small molecular weight, covalently closed, circular, single-stranded RNA molecule that does not encode for a protein and at present only infects plants (83). Both whether these PCV-like mini agents are naked nucleic acids (or encapsulated by the capsid proteins provided by their helper viruses) and whether they are pathogenic to animals remain to be studied further.

Therefore, the in-depth study of PCV2 recombinant viruses and rearrangement molecules will help enrich virological theory, clarify the relationship between PCV2 and PCVAD, and have great practical significance for the evolution of PCV2.

## Author Contributions

LW: writing original draft preparation. LW and KH: writing review and editing. Both authors have read and agreed to the published version of the manuscript.

## Funding

This work was supported by the National Natural Science Foundation of China (Nos. 30972184, 31272574).

## Conflict of Interest

The authors declare that the research was conducted in the absence of any commercial or financial relationships that could be construed as a potential conflict of interest.

## Publisher's Note

All claims expressed in this article are solely those of the authors and do not necessarily represent those of their affiliated organizations, or those of the publisher, the editors and the reviewers. Any product that may be evaluated in this article, or claim that may be made by its manufacturer, is not guaranteed or endorsed by the publisher.
